# Adapting obstetric and neonatal services during the COVID-19 pandemic: a scoping review

**DOI:** 10.1186/s12884-022-04409-4

**Published:** 2022-02-11

**Authors:** Shira Gold, Lauren Clarfield, Jennie Johnstone, Yenge Diambomba, Prakesh S. Shah, Wendy Whittle, Nimrah Abbasi, Cristian Arzola, Rizwana Ashraf, Anne Biringer, David Chitayat, Marie Czikk, Milena Forte, Tracy Franklin, Michelle Jacobson, Johannes Keunen, John Kingdom, Stephen Lapinsky, Joanne MacKenzie, Cynthia Maxwell, Mary Preisman, Greg Ryan, Amanda Selk, Mathew Sermer, Candice Silversides, John Snelgrove, Nancy Watts, Beverly Young, Charmaine De Castro, Rohan D’Souza

**Affiliations:** 1 Division of Maternal-Fetal Medicine, Department of Obstetrics and Gynaecology, Mount Sinai Hospital, University of Toronto, 700 University Avenue, Room 3-908, Toronto, ON M5G 1X5 Canada; 2 Faculty of Medicine, University of Toronto, Toronto, ON Canada; 3 Department of Laboratory Medicine and Pathobiology, Mount Sinai Hospital, Toronto, ON Canada; 4 Department of Paediatrics, Mount Sinai Hospital, Toronto, ON Canada; 5 Department of Family Medicine, Mount Sinai Hospital, Toronto, ON Canada; 6 Department of Family and Community Medicine, Mount Sinai Hospital, Toronto, ON Canada; 7 Department of Nursing, Mount Sinai Hospital, Toronto, ON Canada; 8 Department of Psychiatry, Mount Sinai Hospital, Toronto, ON Canada; 9 Library and Information Services, Mount Sinai Hospital, Toronto, ON Canada

**Keywords:** COVID-19, Pandemics, Coronavirus, Severe acute respiratory syndrome-related coronavirus 2, SARS-CoV-2, Pregnancy, Postnatal care, Neonatology, Perinatology, Perinatal care, Obstetrics, Obstetrical, Maternity, Clinical protocols, Patient care planning, Algorithms, Hospital restructuring, Hospital planning, Health planning guidelines, Quality improvement, Ambulatory care, Simulation training, Personnel management, Medical staff, Medical education, Residency training, Anaesthesia, Ultrasonography

## Abstract

**Background:**

The provision of care to pregnant persons and neonates must continue through pandemics. To maintain quality of care, while minimizing physical contact during the Severe Acute Respiratory Syndrome-related Coronavirus-2 (SARS-CoV2) pandemic, hospitals and international organizations issued recommendations on maternity and neonatal care delivery and restructuring of clinical and academic services. Early in the pandemic, recommendations relied on expert opinion, and offered a one-size-fits-all set of guidelines. Our aim was to examine these recommendations and provide the rationale and context to guide clinicians, administrators, educators, and researchers, on how to adapt maternity and neonatal services during the pandemic, regardless of jurisdiction.

**Method:**

Our initial database search used Medical subject headings and free-text search terms related to coronavirus infections, pregnancy and neonatology, and summarized relevant recommendations from international society guidelines. Subsequent targeted searches to December 30, 2020, included relevant publications in general medical and obstetric journals, and updated society recommendations.

**Results:**

We identified 846 titles and abstracts, of which 105 English-language publications fulfilled eligibility criteria and were included in our study. A multidisciplinary team representing clinicians from various disciplines, academics, administrators and training program directors critically appraised the literature to collate recommendations by multiple jurisdictions, including a quaternary care Canadian hospital, to provide context and rationale for viable options.

**Interpretation:**

There are different schools of thought regarding effective practices in obstetric and neonatal services. Our critical review presents the rationale to effectively modify services, based on the phase of the pandemic, the prevalence of infection in the population, and resource availability.

**Supplementary Information:**

The online version contains supplementary material available at 10.1186/s12884-022-04409-4.

## Introduction

Quality care throughout pregnancy, childbirth and the postnatal period is considered an essential service. The disciplines of obstetrics/midwifery and neonatology, collectively termed perinatology, have decreased maternal and neonatal mortality and morbidity worldwide [1], but the COVID-19 pandemic caused by the Severe Acute Respiratory Syndrome-related Coronavirus 2 (SARS-CoV-2), challenged the safe provision of care [2]. Some early estimates predicted COVID-19 to be the indirect cause of an increase in maternal (8.3–38.6%) and child deaths (9.8–44.7%) in low- and middle-income countries alone [3].

Changes in the provision of care during the pandemic restricted unnecessary physical contact amongst pregnant persons, infants, and healthcare providers and adapts to changing information. Although many academic institutions and national organizations made recommendations on the delivery of perinatal services early in the pandemic, these did not provide enough information for individual institutions to build their own policies, and were made in the absence of strong evidence [4–7]. These limitations made it difficult for clinicians and policy-makers to determine how best to modify their own perinatal services. The objective of this paper is to review the literature, and draw from expert experience at a quaternary care centre, to synthesize and present published recommendations, and where guidelines conflict, provide rationale for selecting the most centre-appropriate modifications.

## Methods

We conducted a scoping review to address our objectives, the checklist of which is presented as Supplementary Data 1. Since the international register for systematic reviews does not register scoping reviews, the protocol was not registered or published. We initially searched Medline, Embase, the Cochrane databases, CINAHL and Scopus from inception until May 14, 2020 using medical subject headings and free-text search terms related to coronavirus infections and pregnancy, and summarized clinical practice recommendations from guidelines of international societies. Prior to submission, we updated targeted searches of general medical and obstetric journals, as well as recommendations from national societies published until December 30, 2020. Our search strategy is presented as Supplementary data 2. Data was charted on forms tested by the research team. One member extracted the data and a second cross-checked for accuracy. In cases of discrepancies, a third investigator independently adjudicated. A list of all data items is presented as Supplementary data 3. We drew upon the expert advice from our hospital, Sinai Health System (SHS), a quaternary referral centre in Toronto, Canada, which was well placed to address the novel coronavirus, based on the experience and lessons learned from the SARS outbreak of 2003, where Toronto was the hardest hit centre outside Asia.

## Results and interpretation

We identified 846 titles and abstracts of which 105 fulfilled eligibility criteria (Supplementary Data 4). These papers mostly included descriptive studies including guidelines, commentaries, expert opinions and committee statements and have been included in our reference list. Since all studies were descriptive, and their scope is clearly outlined in the study titles, study characteristics are not presented separately, but include the entire reference list of this paper. Study findings have been summarized related to organization of services, followed by considerations specific to healthcare providers (HCPs) and health service users (HSUs).

### Organization

#### Leadership and planning

**Fig. 1:**
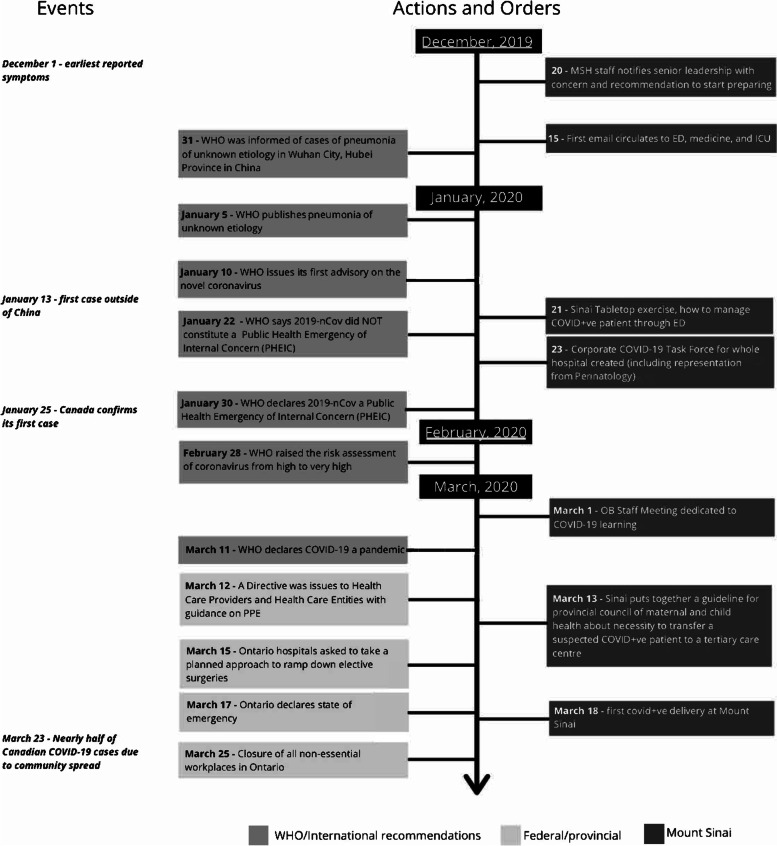
Timeline depicting global events and local response in Toronto with regard to planning for the pandemic

Clear and up-to-date communication from one leadership source at an academic institution is essential to effective implementation of change. Ideally an infectious disease physician and a clinical co-lead should chair the implementation team which includes representation from all clinical and non-clinical departments [8, 9]. In addition to providing oversight and clinical recommendations within the academic setting, the leadership team should liaise with other academic and community hospitals and federal and provincial agencies, to obtain up-to-date evidence and local, context-specific recommendations. Management decisions at the institutional level should be based on local disease prevalence, phase of the pandemic and availability of resources [10]. Within the departments of perinatology, a Steering Committee which includes representation of all HCPs should have virtual meetings as required, to synthesize information, formulate recommendations and disseminate guidance. Early institutional planning is vital and should not await government directives (Fig. 1).

#### Staffing and training considerations

##### Scheduling

Options for staff scheduling changes, based on the phase of the pandemic, local prevalence of cases, and resource availability, include: (1) no change, (2) a formal back-up system (across hospitals, if feasible), in the event of a surge in admissions or reduced staffing from HCPs requiring self-isolation [11, 12], (3) creating HCPs teams always working together and caring for all patients [13], or (4) designating ‘COVID-19 teams’, solely responsible for the care of COVID-19 positive or suspected persons [9, 12, 14–18]. High prevalence areas may benefit from designated care teams/cohorting [19]. HCPs with health or other concerns, should be offered the option of working in lower-risk areas or taking temporary leave [20, 21].

##### Redeployment

Perinatology services that are critically necessary at all times should exempt HCPs from redeployment to intensive care units (ICU)s and infectious disease wards [22]. Cancellation of elective gynaecologic procedures allows increased inpatient capacity [23], and staff availability.

##### Training of staff

Current evidence supports the view that the SARS-CoV-2 virus primarily spreads via droplets, but may be transmitted during aerosol-generating medical procedures (AGMPs) [24]. Training on appropriate donning and doffing of PPE is essential, and most effective through simulation [9, 25]. With adequate PPE and infection control measures, the risk of acquiring an infection within the healthcare setting is low [26].

##### Medical education and residency/fellowship training

Depending on the phase of the pandemic and the ability of healthcare systems to safely cope with increased volumes of extremely high-risk patients, it was suggested that medical students should be removed from clinical care ADDIN EN.CITE [27–29]. This also helped prevent unnecessary exposure of medical students to COVID-19 and conserve PPE. In order to to minimize impact on their education, medical students in several jurisdictions were provided access to print materials and virtual learning tools [27–29]. It may be necessary to suspend subspecialty rotations and deploy trainees to cover emergency perinatology [30], or other emergency services. Reducing trainee work hours could facilitate the creation of a backup pool supporting trainees who are ill or self-isolating. In-person educational activities and non-essential clinical activities should be cancelled [30, 31] or moved online [32, 33]. Fellowship training programs that recruit post-residency trainees could continue with minimal changes, with fellows providing virtual and in-person clinics, in-house team call, and clinical service on the wards.

##### Health care professionals’ (HCPs) wellness

Universal screening of HCPs at the hospital/clinic entrance should be considered; those screening positive should be tested and self-isolate until results are available or for 14-days. Monitoring symptoms of COVID-19 [34] include measuring temperatures twice daily, having a dedicated clinic to assess HCPs with symptoms [35], and ensuring 14-days of self-isolation for those exposed to COVID-19 without adequate PPE [34, 36, 37]. HCPs are also at increased risk for psychological distress and mental health problems during pandemics [38]. Recommendations for promoting psychosocial wellness include recognition of efforts, creating back-up schedules to avoid fatigue [9], providing discussion forums to raise concerns [38], and the availability of dedicated psychiatrists and counsellors to provide resilience coaching and support.

#### Care of the pregnant person

##### Screening and testing

Universal screening via telephone, for symptoms and risk factors the day prior to appointments, and again upon entry into a clinical setting, is an effective risk-reduction method [4, 9, 28, 29, 34, 39–42]. If deferral of appointments for screen-positive persons is not possible, protocols as described in Supplementary Data 5 should be implemented. Testing policies could vary from universal testing of all HSUs to testing only those that are screen-positive, depending on community prevalence of COVID-19, testing capacity, turnaround time for test results and the availability of PPE for all HSUs and HCPs under investigation while awaiting results [43–46].

##### Antenatal care – ambulatory settings

**Fig. 2:**
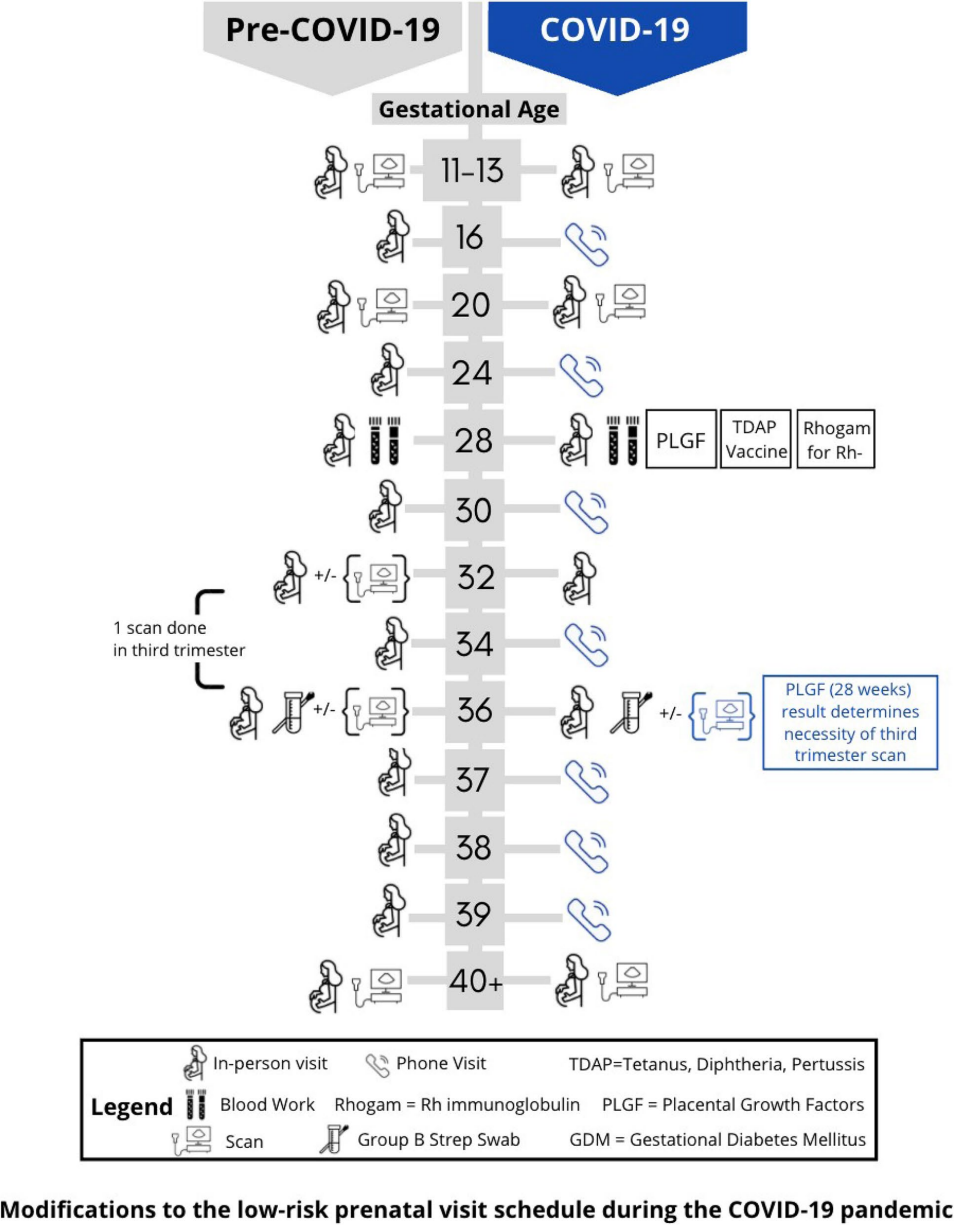
Modifications to the low-risk prenatal clinic schedule during the COVID-19 pandemic

**Fig. 3:**
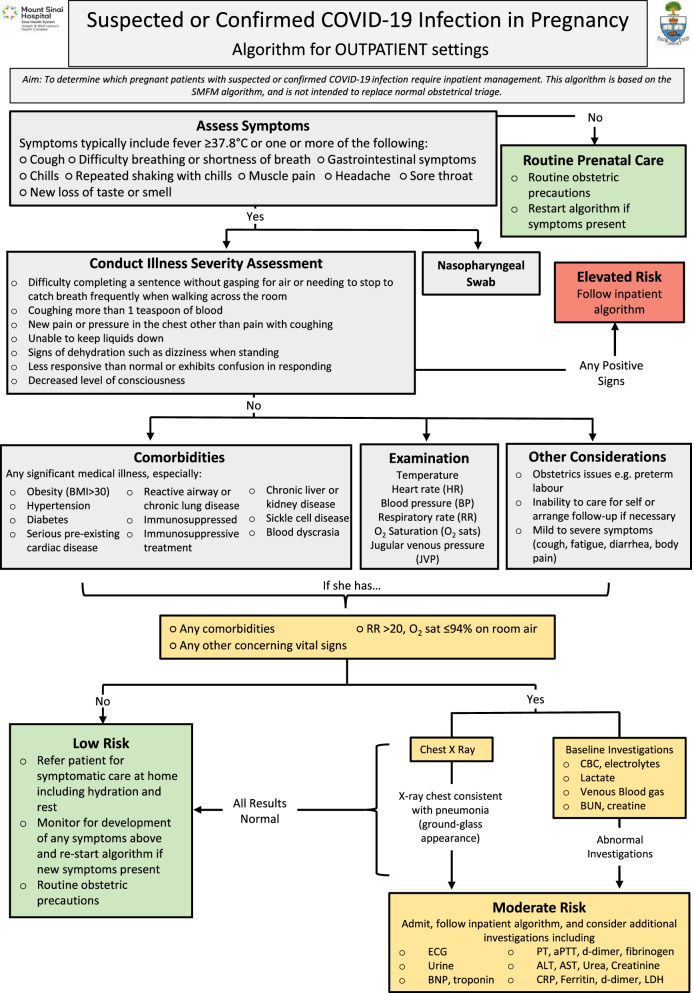
Algorithm for the management of persons with suspected or confirmed COVID-19 infection in pregnancy in the outpatient setting

Centres should have systems that limit physical exposure between and among HSUs and HCPs. Telephone and videoconferencing can effectively limit the number of in-person visits, and can be scheduled to coincide with routine blood or ultrasound tests [4, 28, 36, 42, 47–54]. Any modifications to care, as illustrated in Fig. 2, should consider limitations of virtual care, which include barriers to access, language skills, and impaired HSU-HCP relationships [53]. A summary of COVID-specific considerations at in-person antenatal visits for low-risk pregnancies is presented in Table 1. Basic principles for the management of high-risk pregnancies include individualization of care plans and 24-h access to a telephone line in case of emergencies and specific considerations are discussed in Table 2. Specialized Ambulatory Units such as obstetrical day units, which provide non-urgent in-person services including administration of antenatal corticosteroids, blood pressure assessments, blood work and non-stress tests; and obstetrical triage may continue to offer services, and possibly expand their scope to limit hospitalization, with strict protocols/algorithms (Fig. 3) and designated rooms for screen-positive persons. Risk-reduction strategies during in-person visits include diligent hand and surface hygiene and wearing of surgical masks by HCPs [34, 36, 39, 55] and symptomatic [9, 20, 28, 34, 40] or all HSUs [28, 39, 53, 55, 56]. Screen-positive HSUs should wear a mask, wait in a designated area prior to assessment [9, 40], and enter an assessment room along a designated route guided by a HCP in full droplet- and contact PPE [53]. Paper charts should be avoided in the assessment room, and there should be clear signage describing the necessary cleaning protocols. Strategies to minimize contact between and among HSUs and HCPs include assessment of vital signs by physicians in physician-led units to avoid additional contact with a nurse at each visit [40], creation of distanced waiting areas [50], and increasing time between appointments [29]. Physical space modifications include ensuring that triage/screening areas are separate and well-ventilated, incorporating plexiglass barriers to triage settings, placing chairs in waiting rooms six feet apart, and providing hand hygiene stations [9, 28, 29, 40, 55]. Special clinics/ hospitals could be designated for providing antenatal care to COVID-19-positive or suspected persons in high-prevalence areas [11, 39, 50, 57]. Centres should have contingency plans if case numbers increase (Supplementary data 6). Decisions regarding the presence of partners/ support persons during antepartum appointments should be based on the patient volume at each centre, the ability to follow physical distancing protocols, while safely providing in-person care to the birthing parent and emerging evidence on the impact of restrictions on maternal physical and mental health, preterm birth and stillbirths [58–61]. Pregnancy and parenting education classes could be conducted online, if possible. While making these decisions, the human impact of these restrictions need to be considered.

**Table 1:** Considerations at in-person antenatal visits for low-risk pregnancies

**Initial visit**	Determine eligibility for low-risk modified schedule
**Aneuploidy screening**	• Continue to offer • Preferred option - first trimester screen (FTS) which includes ultrasound assessment of nuchal translucency, twin chorionicity, fetal anomalies [62] and pregnancy dating. • *[For COVID-19 positive or suspected persons, defer for 2 weeks if still in the appropriate window, or to screen with non-invasive prenatal testing (NIPT) or second trimester maternal serum screening]* [51].
**Anatomical ultrasound**	• Continue to offer; prioritize over other obstetrical ultrasounds [51]. • Discourage early anatomical scans (< 18 weeks) and encourage later scans (closer to 22 weeks) to reduce risk of suboptimal views and need for repeat scans.
**Screening for gestational diabetes mellitus (GDM)**	• Continue to offer • Avoid protocols involving longer wait times and multiple contacts between care providers and patients for blood draws. • Consider alternate screening strategies such as measuring glycosylated hemoglobin (HbA1c) and random plasma glucose (RPG) through a single blood draw at the 28-week visit, and diagnosing GDM if HbA1c ≥5.7% or RPG ≥11.1 mmol/L [63].
**Third trimester visits**	• Consider modified antenatal schedule (Fig. 2). • Encourage self-monitoring of blood pressure, blood glucose, uterine height and fetal movements, if possible [42, 47, 48, 50, 52].
**Ultrasound scans for fetal growth and wellbeing**	• Adhere to ISUOG consensus statement [51]. • Consider discussing ultrasound findings via telephone [29] • (Experimental) – consider using 28-week placental growth factor testing [64] to determine those in whom routine third-trimester ultrasound scans can be avoided. • *[No strong evidence to suggest 2-to-4-weekly ultrasound assessments* [18, 34, 39, 55] *for those with COVID-19, since unlike with Severe Acute Respiratory Syndrome* [53], *there is no conclusive data suggesting an association between COVID-19 infection fetal growth restriction].*
**Group B Streptococcal (GBS) Screen**	• Continue to offer, but consider self-administration by pregnant person, timed with a scheduled in-person visit between 35 and 37 weeks (Fig. 2). • *[In those with confirmed or suspected COVID-19, testing could either be delayed by up to 14 days or intrapartum antibiotic prophylaxis could be administered using a risk-factor-based approach.]* [34, 37]

*[Italicized text]* indicates suggestion for those with suspected or confirmed COVID-19

*ISUOG* International Society of Ultrasound in Obstetrics & Gynecology

**Table 2:** Considerations at in-person antenatal visits for high-risk pregnancies during the pandemic

**Genetics**	• Continue to offer; genetic testing and diagnostic procedures are considered essential, but not emergent [65–67]. Consider deferring non-pregnant consults, unless a timely appointment is necessary, such as in the case of advanced maternal age. • *[Defer by 2 weeks if possible in those who are COVID-19 positive or suspected* [65, 67, 68]*].* • *[Amniocentesis, with a lower risk of vertical transmission from intra-amniotic bleeding and disruption of the feto-maternal barrier, has a theoretical advantage of over chorionic villi sampling (CVS)* [65–67].*]* • To minimize in-person contact, consider creation and dissemination of PowerPoint presentations on genetic conditions, screening and diagnostic procedures, pregnancy termination options and contraceptive services in multiple languages.
**Fetal disorders**	• Given the reliance on ultrasound, virtual care is not feasible in fetal medicine clinics. Consider organizational changes to reduce in-person contact including history-taking by senior personnel via virtual platforms prior to the in-person appointment, ultrasound scans by experienced staff during the in-person visit and virtual counselling following the appointment.
**Fetal Therapy**	• Fetal therapies should not be considered elective, and life-preserving procedures should continue, with appropriate modifications, within the context of local resources [65, 66]. At our hospital, which is home to the Ontario Fetal Centre, the largest and most advanced fetal therapy centre in Canada [69], life-saving procedures including fetal blood transfusion, fetoscopic placental laser ablation and amnioreduction for twin-to-twin transfusion syndrome, and shunting procedures continued to be available. The resource-intensive fetal myelomeningocele closure program which was initially halted, soon resumed given the low disease prevalence in Toronto. • *[Procedures should be deferred if safely possible in those with confirmed or suspected COVID-19]*
**Pregnancy termination**	• Abortion care is considered an essential service, due to its time-sensitive nature and implications to a person’s life, health, and well-being [70].
**Preterm birth**	• Suggested modifications to the management of those at risk for preterm birth include initiation of cervical length screening for high-risk pregnancies at 16 weeks, with discharge from clinic if stable cervical length at 18 and 20 weeks [51], delaying ultrasound scans in COVID-19 positive or suspected and starting progesterone instead [51], and trans-abdominal vs. transvaginal measurement of cervical length [55]. Since these recommendations are based on limited evidence, in our clinic, we continued two-weekly transvaginal cervical length measurement, between 18 and 28 weeks, as was the case prior to the pandemic. Both elective and rescue cerclage continued to be offered, given their time-sensitive nature.
**Medical Disorders**	• Consider reducing frequency of inpatient visits, through provision of equipment to monitor blood pressure, blood sugar and fetal movements, as required.

*[Italicized text]* indicates suggestion for those with suspected or confirmed COVID-19

##### Antenatal care - inpatient settings

**Fig. 4:**
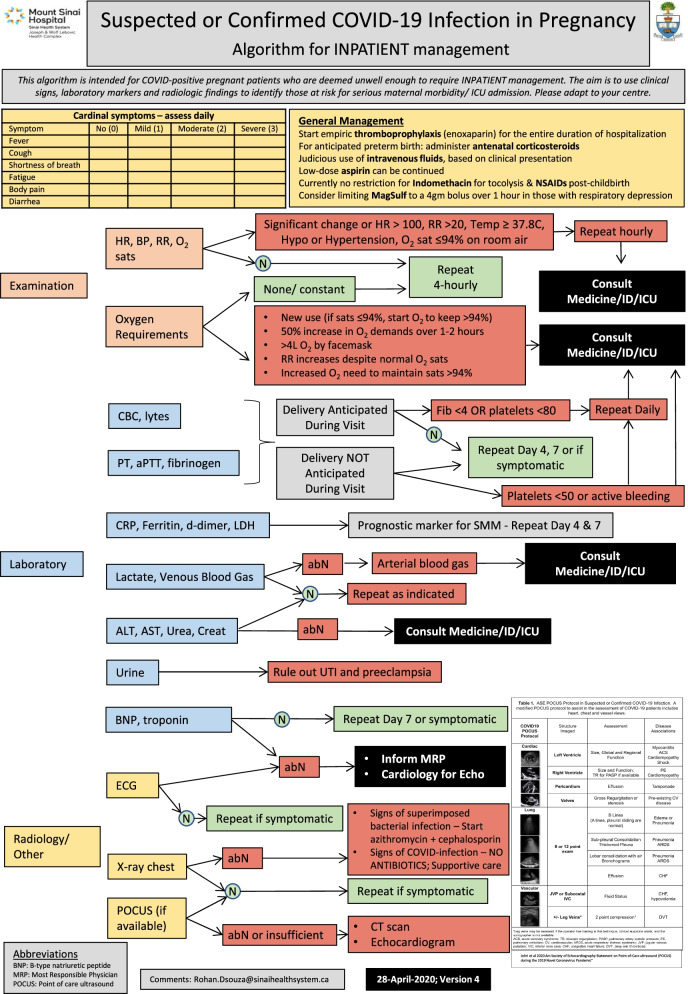
Algorithm for the management of persons with suspected or confirmed COVID-19 infection in pregnancy in the inpatient setting

A positive COVID-19 result is not an indication for hospital admission or transfer to a higher centre; inpatient management should only be considered when medically indicated [21, 42, 71]. Those admitted for COVID-unrelated concerns, should be monitored daily for development of COVID-19 symptoms, and those admitted with suspected or confirmed COVID-19 should be systematically assessed for disease progression using algorithms such as the one presented in Fig. 4. In high-prevalence areas, sequestration of HSUs with suspected and confirmed COVID-19 in isolated wards [11, 28, 34, 37, 39, 72], management by specific HCPs [9], or redirection to designated hospitals may be considered [39, 57]. These policies need regional cooperation. In addition to structural modifications to inpatient units, measures to limit HSU-HCP contact include limiting blood-draws and avoidable assessments, care by the senior-most and fewest numbers of HCPs, and using virtual platforms for handovers. An evidence-based approach to the use of routine and experimental medications is described elsewhere [73, 74].

##### Intrapartum care

COVID-19 is not an indication for inducing labour or performing a caesarean [4, 6, 13–15, 21, 34, 36, 39, 41, 42, 50, 75–77]. Scheduled births may be delayed in COVID-19 positive and suspected HSUs to enable confirmatory testing [15], allow time for recovery, optimize one’s respiratory status [78] and reduce the risk to themselves, HCPs and neonates [4, 6, 41]. Timing of birth must consider the HSU’s clinical status, gestational age and fetal condition [4, 6, 34, 41, 42]. While medically-indicated labour inductions should continue uninterrupted [15, 36, 78], decisions on non-urgent indications must be based on a risk-benefit assessment that includes duration of hospitalization, contact with HCPs, resource utilization and the perceived risk of continuing the pregnancy. Outpatient cervical ripening is safe and effective [79], and has the advantage of reducing the length of hospital stay [15]. Telephone-screening for symptoms the day before scheduled inductions or planned caesareans, as well as upon arrival, is recommended. While hospital-births are considered safest for those with confirmed or suspected COVID-19, decisions regarding homebirths for non-infected individuals, to minimize contact with HCPs should depend on locally-available infrastructure and regional/cultural acceptance [53]. Suggested modifications to protocols for hospital births including visitor policies are presented in Table 3. Decisions around labour analgesia are often personal, with considerable regional variation. Regional (epidural) analgesia has been recommended early in labour to avoid exacerbation of respiratory symptoms secondary to labour pain [15, 17, 25, 34–36, 76, 80, 81], and the need for general anaesthesia in case of an emergency caesarean. Neuraxial anaesthesia (spinal or epidural) is the preferred modality for caesareans [11, 15, 25, 34, 35]. Widespread use of epidurals could increase the incidence and severity of intrapartum pyrexia, which could result in designating a HSU as a suspected case of COVID-19, requiring increased use of PPE [80]. Some organizations have advocated for suspending the use of nitrous oxide for labour analgesia, because of possible aerosolization [15, 36, 56, 81], while others suggest its use may be acceptable with precautions such as a single-use microbiological filter [13, 25, 35]. Hydrotherapy (water births) was disallowed by certain groups due to possible presence of SARS-Cov-2 in feces [34, 42, 53].

**Table 3:** Modifications to protocols for labour and childbirth

**Airborne infection isolation rooms**	• AGMPs can theoretically cause aerosolization of SARS-CoV-2, and therefore the use of airborne infection isolation rooms for the care of COVID-19 positive or suspected parturients is recommended if an AGMP is being performed [11, 15, 21, 35, 39, 42, 78, 82]. • *[If available, one operating room with negative pressure and an anteroom should be marked exclusively for those with confirmed or suspected COVID-19, that needed emergency surgery.]*
**Visitors and birth-attendants**	• Decisions should consider disease prevalence and regional/ cultural norms, the life-altering nature of the birthing experience and reports of increased stress and anxiety for pregnant persons with restrictive visitor policies [83, 84]. Options include (1) no visitors [35, 83], (2) one visitor who must leave following childbirth [15, 25, 34, 36, 72], and (3) one visitor for the duration of admission [4, 6, 39, 41, 46, 84, 85]. • All visitors should be screened and allowed only if they screen negative [4, 15, 46, 56]. • More accommodating visitor policies can be carefully introduced in the context of the available literature, which does not endorse support persons as a route of transmission of COVID-19 in hospitals [86]. • *[Several guidelines recommend no visitors for parturients who are COVID-19 positive or suspected* [4, 15, 46, 78].*]*
**PPE for care providers**	• For vaginal births, routine practice should include hand hygiene, wearing of gloves, protective eyewear and gowns [15, 40, 55, 56, 87] • *[In addition to routine measures, Droplet and Contact Precautions are recommended for care of known or suspected COVID-19 persons for non-aerosolizing medical procedures, such as the management of the first stage of labour* [4, 6, 41, 53, 88]. *Since it is unclear whether forceful exhalation in the second stage of labour has the potential to generate aerosols, most guidance suggest using N95 respirators for vaginal birth of a COVID-19 positive or suspected person, if available* [34, 35, 55, 89, 90]*].*
**The use of masks by parturients**	• Decisions depend, to some extent, on the universality of testing prior to admission. The universal use of masks by all parturients throughout admission [18, 39, 55, 56] may not be necessary, although it should be considered during transfers [50], and in all public areas. This protects others, while ensuring the comfort of the parturient during the extended stay and in active labour. • [Wearing of masks by those positive or suspected of COVID-19 should be encouraged [6, 40–42].]
**Intrapartum fetal monitoring**	• Continue as indicated by local policy and clinical indication. • *[Continuous electronic fetal monitoring has been recommended for symptomatic parturients with confirmed or suspected COVID-19, but not for asymptomatic or mild cases* [53].*]*
**Management of the second stage of labour**	• Continue according to local policy and clinical indication. • *[Although operative vaginal delivery has been recommended to reduce the duration of active pushing and forceful exhalation that could risk spread of infection* [18, 34, 42, 56, 78], ,*there is no clinical justification for this practice, unless the parturient has considerable respiratory distress.]*
**Emergency caesarean deliveries**	• Although the indications for emergency caesareans remain unchanged, consideration must be given to additional time required for donning PPE and the risk posed by intubation at the time of dire emergencies [34, 76]. • Involvement of the senior most anaesthesia and obstetric staff could minimize complications and reduce the need for repeat operation [76]. • Consider avoiding staples for skin closure, to reduce additional follow-up for their removal [50].

*[Italicized text]* indicates suggestion for those with suspected or confirmed COVID-19

*AGMP* Aerosol-generating medical procedure, *SARS-CoV-2* Severe Acute Respiratory Syndrome-related coronavirus 2, *PPE* Personal protective equipment

##### Postpartum care

Multiple transfers between birthing and recovery units should be avoided and the duration of postpartum hospitalization should be reduced where possible [15, 50, 78, 81].

Although not ideal, depending on a local risk-benefit assessment, group breastfeeding and discharge classes may be replaced by instructive videos. One-on-one care should be provided for those that require additional breastfeeding support prior to discharge. For those that meet pre-specified criteria (Supplementary data 7), early discharge and screening at home within 24–36 h of birth by midwives should be considered [9, 15, 57]. Those requiring blood draws or wound care could be assessed in Postnatal Ambulatory Care clinics and the scheduled six-week postpartum visit may be conducted virtually.

##### Care of the critically ill pregnant person

Pregnant persons with COVID-19 are at risk of life-threatening complications, particularly acute respiratory failure, shock and thromboembolic disease, requiring review by a critical care rapid response team and sometimes ICU admission [91]. Early warning scores can indicate escalation through detection of worsening oxygen saturation, increasing respiratory rate, and decreased level of alertness [92]. The ICU should have equipment and drugs for vaginal or caesarean birth and for neonatal resuscitation. A nearby location should be identified for potential neonatal resuscitation, allowing airborne precautions. Although ICU management is not different in the pregnant person, and there are no data to suggest an alteration to usual ventilatory approaches, airway management requires a higher degree of skill and prone positioning may be more difficult [93]. Although delivery may not always result in significant improvement of respiratory distress [94, 95], this may improve maternal oxygenation when conservative measures have failed [13, 14, 21, 34, 36, 50, 75, 76].

##### Health service user’s (HSU) wellness

There has been a considerable increase in self-reported depression and anxiety during the pandemic [96], possibly due to isolation, job and financial insecurity, intimate partner violence and reduction in support systems [42, 53, 88]. HCPs should ask about HSU’s mental health during every encounter [4, 53]. At our centre, referral to a perinatal mental health team, composed of social workers and perinatal psychiatrists, can be made for any mental health concerns in pregnancy. Our obstetric and psychiatry teams developed weekly interactive pregnancy-specific webinars to discuss adaptations to care and mental health topics.

##### Vaccination

Pregnant women were initially excluded from vaccine trials and safety data was therefore limited. Therefore, the UK had initially recommended against vaccination in pregnant persons or those planning to conceive within 3 months, but did not describe vaccination as an indication for termination [5]. The US and Canada, cautiously supported vaccination, particularly for those at high risk of infection and/or morbidity [7, 97]. Now, it is universally recommended by all national organizations for all pregnant persons to be vaccinated by COVID-19 during pregnant women [4–7]. Although vaccine-elicited SARS-CoV-2 antibodies have been isolated in neonatal cord blood and in breast milk, however, the degree of passive immunity is not confirmed [53].

#### Neonatal care

**Fig. 5:**
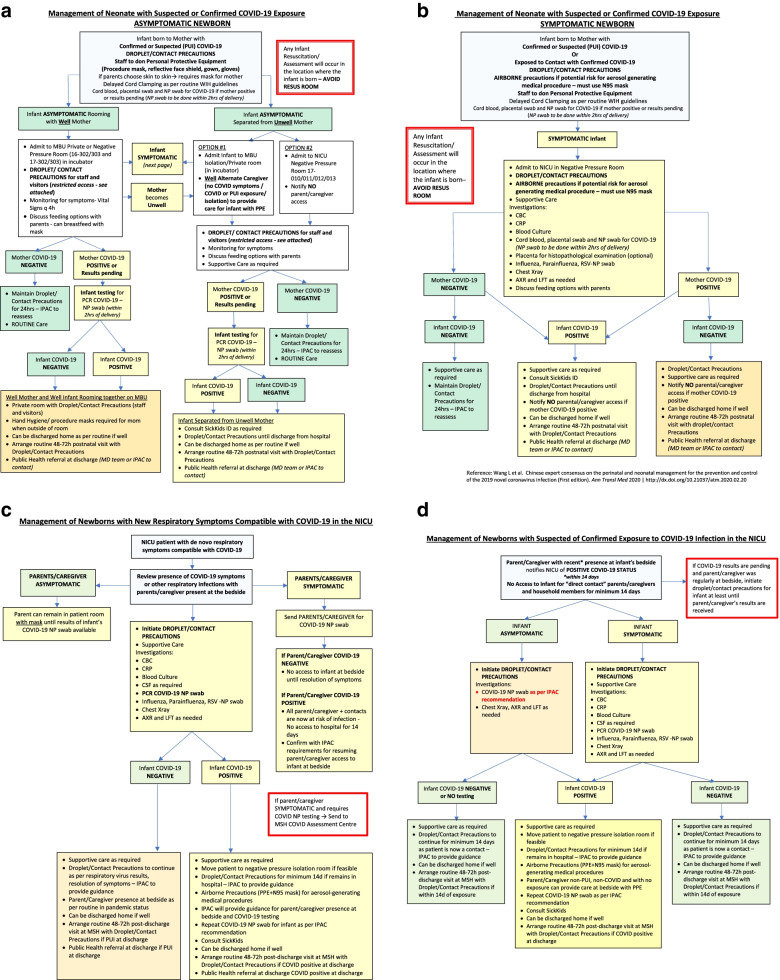
Neonatal Unit Algorithms

Care for infants during the COVID-19 pandemic must carefully balance the risk of COVID-19 exposure with the benefits of infant-parent bonding. The contentious issues that influence care of the neonate are described in Table 4. In addition, examples of modified clinical care pathways for management of symptomatic neonates or those born to mothers with confirmed or suspected COVID-19 are detailed in Fig. 5. In the absence of adequate PPE and individual rooms for neonates, physical changes to the Neonatal Intensive Care Unit include moving monitors to doorways of high-risk infant rooms or using central monitoring, and using long-tubing intravenous lines [14]. Neonatal follow-up after discharge from hospital should be conducted using virtual platforms wherever possible.

**Table 4:** Neonatal care policies (after Chandrasekaran et al) [77]

**Transplacental transmission**	• Although the presence of the angiotensin-converting enzyme 2 receptor used by SARS-CoV-2 in the placenta [21, 98], makes transplacental transmission plausible, to date, this has only been confirmed in a minority of cases [99–102].
**Delayed cord clamping (DCC)**	• Continue in accordance with unit policies. Benefits of DCC include increased haemoglobin and iron stores in term infants, and improved transitional circulation, better establishment of red blood cell volume, decreased need for blood transfusion, and lower incidence of necrotizing enterocolitis and intraventricular haemorrhage in preterm infants [103]. • *[In COVID-19 positive or suspected mothers, some groups recommend immediate cord clamping* [13, 15, 21, 36, 39, 42, 78, 82], *while others encourage DCC* [4, 77, 88]. *Shared decision-making on risks and benefits is recommended]*
**Neonatal resuscitation**	• Drying, tactile stimulation, and assessment of heart rate are non-aerosol-generating, while suction or endotracheal intubation or medication instillation, are considered to be AGMPs, and therefore require donning of PPE by the resuscitation team [21]. • *[For neonates born to COVID-19 positive or suspected mothers, resuscitation should be carried out in a separate room, and, if not feasible, at a distance of 6 m apart with a physical barrier between mother and baby, preferably in an isolette with a hood* [77].*]* • *[It is also recommended that neonates born to persons with active COVID-19 infections are washed as soon as possible after birth in order to reduce transmission risk* [21, 77].*]* • *[Although it has been suggested that designated resuscitation teams attend all COVID-19 positive or suspected births, in order to minimize exposure to care providers and uninfected persons* [77], *this may not be necessary in areas of low prevalence and neonatologists could only attend births where the neonate is likely to require resuscitation or early neonatal care.]*
**Skin-to-skin**	• Continue in non-infected individuals, since this practice has numerous benefits including decreased postpartum maternal anxiety, decreased depression in the first year postpartum, increased uterine tone with decreased bleeding, and improved weight gain and sleep quality in the newborn [88]. • *[Although skin-to-skin contact between a COVID-19 positive or suspected parent and a neonate has been discouraged by many* [13, 15, 21, 34, 35, 39, 42, 76, 81, 82, 104], *due to the risk of postnatal transmission, this may still be possible following shared decision-making in asymptomatic individuals, with mask-wearing and appropriate hand and breast hygiene.]*
**Breastfeeding**	• Continue to offer in non-infected persons. • *[For those with suspected or confirmed COVID-19, the risk of transmission of SARS-CoV-2 to infants is more likely to be* via *respiratory droplets while feeding as opposed to transmission* via *breastmilk* [105]. *Options include:* (1) *no breastfeeding and no feeding of expressed breastmilk* [39, 81], (2) *no breastfeeding but permitting the feeding of expressed breastmilk to infant* [18, 82, 106], (3) *direct breastfeeding* [53, 57, 105, 107]. *Some groups specify that a mother with asymptomatic or mild disease may breastfeed, but if severely or critically ill only expressed breastmilk should be used* [21, 34]*. Given that these recommendations are based on limited evidence, decisions should be individualized, and consider all pros and cons. While not breastfeeding, neonates should be at least 6 ft away from infected mothers, and mothers should be masked at all times. Those not comfortable with the risks of breastfeeding should be encouraged to express breastmilk.]*
**Separation or co-location of mother and baby**	• *[Many groups recommend separation of mother and baby in the case of confirmed or suspected COVID-19* [15, 18, 39, 50, 76, 81, 108, 109], *while others permit rooming-in for infants with precautionary measures in place* [34, 53, 57, 88]. *Shared decision-making is encouraged, if the mother is not too unwell to care for the baby.]*
**Neonatal testing**	• There is considerable variation in testing of babies born to unaffected mothers, and decisions should be based on local-prevalence, availability of testing and local policies. Some groups tested all babies admitted to the NICU [110], while others recommended against it as this often resulted in false negative results [53]. • *[Testing of neonates born to mothers with confirmed or suspected COVID-19, regardless of maternal symptoms, at approximately 24 h of age is widely practiced* [21, 57, 81, 104, 111]. *If initial test results are negative, or not available, repeat testing is recommended at 48 h of age* [21, 104]. *Placental and cord blood samples may be collected and tested by swab and histopathology in order to better understand transplacental transmission.]*
**Visitor policies**	• Decisions should be individualized based on local prevalence, condition of the neonate and resource-availability. Modifications to visitor policies included limiting visitors to one parent at a time [15, 57, 110], with some groups specifying mothers only [57], or to none at all [83, 110]. • If screen-negative parents are permitted to visit, consider restricting movement in and out of the NICU’

*[Italicized text]* indicates suggestion for those with suspected or confirmed COVID-19

*NICU* Neonatal intensive care unit, *AGMP* Aerosol-generating medical procedure, *SARS-CoV-2* Severe Acute Respiratory Syndrome-related coronavirus 2, *PPE* Personal protective equipment

#### Research

Physical distancing recommendations require the suspension of many clinical and basic science research activities [30], in favour of COVID-19-related research in diagnostic, therapeutic and preventative approaches, and their effects on pregnancy and the neonatal period [60, 112]. Resumption of routine research activity should include careful planning, staggered work hours, smaller research teams and virtual lab meetings [30]. While data on maternal and fetal effects from COVID-19 are being gathered by registries, pregnant persons continue to be excluded from clinical trials, which could result in their failure to receive treatments due to unsubstantiated concerns [112].

## Conclusions

The provision of high quality and evidence-based perinatal care must remain a priority, even in the face of a pandemic. Despite the limitations, which include reliance on descriptive studies and a lack of high-quality evidence, our scoping review presents a practical framework that can guide clinicians, administrators, educators, and researchers in their efforts to effectively modify services, based on the phase of the pandemic, the prevalence of infection in the population, and resource availability. Although the provision of a detailed critical analysis of each recommendation was out of reach of this scoping review; it provides the available options, rationale behind them, and implementation strategies to individualize an institution’s pandemic response. As with any guidance, these recommendations need to be considered in the light of their impact on the short- and long-term physical and psychological wellness of families, society, medical students, trainees and healthcare providers. At each stage of the pandemic, policymakers should perform risk-benefit analyses to determine the appropriateness of recommendations, while considering the evolving evidence and feedback.

## Supplementary Information

**Additional file 1: Supplement 1.** Prisma Scoping Review Checklist. **Supplement 2.** Search Strategy. **Supplement 3.** List of Data Items. **Supplement 4.** Prisma Flow chart. **Supplement 5.** Ambulatory Visit of a Confirmed or Suspected Case of COVID-19. **Supplement 6.** COVID-19 Antenatal Care Clinic. **Supplement 7.** Low-Risk Inclusion Criteria for Early Discharge, less than 24 h after birth.

## Data Availability

Data sharing is not applicable to this article as no datasets were generated or analyzed during the current study.
