# Supplementary material for: Adapting obstetric and neonatal services during the COVID-19 pandemic: a scoping review
Source: BMC Pregnancy Childbirth. 2022 Feb 11;22:119. doi: 10.1186/s12884-022-04409-4 (PMC8840792; doi:10.1186/s12884-022-04409-4)
Supplement: Supplementary file 1 — Additional file 1: Supplement 1. Prisma Scoping Review Checklist. Supplement 2. Search Strategy. Supplement 3. List of Data Items. Supplement 4. Prisma Flow chart. Supplement 5. Ambulatory Visit of a Confirmed or Suspected Case of COVID-19. Supplement 6. COVID-19 Antenatal Care Clinic. Supplement 7. Low-Risk Inclusion Criteria for Early Discharge, less than 24 h after birth. [file 12884_2022_4409_MOESM1_ESM.pdf]

Supplement 1: Prisma Scoping Review Checklist

Supplement 2: Search Strategy

Supplement 3: List of Data Items

Supplement 4: Prisma Flow chart

Supplement 5: Ambulatory Visit of a Confirmed or Suspected Case of COVID-19

Supplement 6: COVID-19 Antenatal Care Clinic

Supplement 7: Low-Risk Inclusion Criteria for Early Discharge, less than 24 hours after birth

Preferred Reporting Items for Systematic reviews and Meta-Analyses extension for Scoping Reviews (PRISMA-ScR) Checklist

SECTION	ITEM	PRISMA-ScR CHECKLIST ITEM	REPORTED ON PAGE #
**TITLE**			
Title	1	Identify the report as a scoping review.	1
**ABSTRACT**			
Structured summary	2	Provide a structured summary that includes (as applicable): background, objectives, eligibility criteria, sources of evidence, charting methods, results, and conclusions that relate to the review questions and objectives.	4-5
**INTRODUCTION**			
Rationale	3	Describe the rationale for the review in the context of what is already known. Explain why the review questions/objectives lend themselves to a scoping review approach.	6
Objectives	4	Provide an explicit statement of the questions and objectives being addressed with reference to their key elements (e.g., population or participants, concepts, and context) or other relevant key elements used to conceptualize the review questions and/or objectives.	6
**METHODS**			
Protocol and registration	5	Indicate whether a review protocol exists; state if and where it can be accessed (e.g., a Web address); and if available, provide registration information, including the registration number.	6-7
Eligibility criteria	6	Specify characteristics of the sources of evidence used as eligibility criteria (e.g., years considered, language, and publication status), and provide a rationale.	6-7
Information sources*	7	Describe all information sources in the search (e.g., databases with dates of coverage and contact with authors to identify additional sources), as well as the date the most recent search was executed.	6-7
Search	8	Present the full electronic search strategy for at least 1 database, including any limits used, such that it could be repeated.	supplement1
Selection of sources of evidence†	9	State the process for selecting sources of evidence (i.e., screening and eligibility) included in the scoping review.	6-7
Data charting process‡	10	Describe the methods of charting data from the included sources of evidence (e.g., calibrated forms or forms that have been tested by the team before their use, and whether data charting was done independently or in duplicate) and any processes for obtaining and confirming data from investigators.	7
Data items	11	List and define all variables for which data were sought and any assumptions and simplifications made.	supplement XX
Critical appraisal of individual sources of evidence§	12	If done, provide a rationale for conducting a critical appraisal of included sources of evidence; describe the methods used and how this information was used in any data synthesis (if appropriate).	NA
Synthesis of results	13	Describe the methods of handling and summarizing the data that were charted.	

SECTION	ITEM	PRISMA-ScR CHECKLIST ITEM	REPORTED ON PAGE #
**RESULTS**			
Selection of sources of evidence	14	Give numbers of sources of evidence screened, assessed for eligibility, and included in the review, with reasons for exclusions at each stage, ideally using a flow diagram.	7 (Lauren/ Shira will send)
Characteristics of sources of evidence	15	For each source of evidence, present characteristics for which data were charted and provide the citations.	7, 16-30
Critical appraisal within sources of evidence	16	If done, present data on critical appraisal of included sources of evidence (see item 12).	NA
Results of individual sources of evidence	17	For each included source of evidence, present the relevant data that were charted that relate to the review questions and objectives.	7-15
Synthesis of results	18	Summarize and/or present the charting results as they relate to the review questions and objectives.	7-15
**DISCUSSION**			
Summary of evidence	19	Summarize the main results (including an overview of concepts, themes, and types of evidence available), link to the review questions and objectives, and consider the relevance to key groups.	7-15
Limitations	20	Discuss the limitations of the scoping review process.	15
Conclusions	21	Provide a general interpretation of the results with respect to the review questions and objectives, as well as potential implications and/or next steps.	16
**FUNDING**			
Funding	22	Describe sources of funding for the included sources of evidence, as well as sources of funding for the scoping review. Describe the role of the funders of the scoping review.	2

JBI = Joanna Briggs Institute; PRISMA-ScR = Preferred Reporting Items for Systematic reviews and Meta-Analyses extension for Scoping Reviews.
* Where *sources of evidence* (see second footnote) are compiled from, such as bibliographic databases, social media platforms, and Web sites.
† A more inclusive/heterogeneous term used to account for the different types of evidence or data sources (e.g., quantitative and/or qualitative research, expert opinion, and policy documents) that may be eligible in a scoping review as opposed to only studies. This is not to be confused with *information sources* (see first footnote).
‡ The frameworks by Arksey and O'Malley (6) and Levac and colleagues (7) and the JBI guidance (4, 5) refer to the process of data extraction in a scoping review as data charting.
§ The process of systematically examining research evidence to assess its validity, results, and relevance before using it to inform a decision. This term is used for items 12 and 19 instead of "risk of bias" (which is more applicable to systematic reviews of interventions) to include and acknowledge the various sources of evidence that may be used in a scoping review (e.g., quantitative and/or qualitative research, expert opinion, and policy document).

From: Tricco AC, Lillie E, Zarin W, O'Brien KK, Colquhoun H, Levac D, et al. PRISMA Extension for Scoping Reviews (PRISMA-ScR): Checklist and Explanation. Ann Intern Med. ;169:467–473. doi: 10.7326/M18-0850

MAY 15, 2020 SEARCH RUN

Medline Final Search:

Database(s): Ovid MEDLINE(R) ALL 1946 to May 14, 2020
Search Strategy:

#	Searches	Results
1	exp Coronavirus/	13719
2	exp Coronavirus Infections/	12380
3	(coronavirus* or corona virus* or corona virinae* or coronavirinae* or OC43 or NL63 or 229E or HKU1 or HCoV* or covid* or ncov* or coV or sars-cov* or sarscov* or Sars-coronavirus* or Severe Acute Respiratory Syndrome Coronavirus* or 2019-ncov or 2019-novel CoV or SARS-like coronavirus*).mp.	28676
4	((novel or new or nouveau) adj2 pandemi*2).mp.	904
5	((pneumonia or sars*).mp. or exp pneumonia/) and Wuhan.mp.	949
6	(COVID-19 or severe acute respiratory syndrome coronavirus 2).os,ps,rs,ox,px,rx,nm.	2888
7	or/1-6	34565
8	exp Pregnancy/	888385
9	exp Pregnancy Complications/	423032
10	exp Pregnancy Outcome/	73132
11	exp Obstetrics/	22538
12	exp Breast Feeding/	37387
13	exp Maternal Health Services/	48885
14	exp Fetus/	156690
15	exp Fetal Therapies/	3990
16	exp Fetal Monitoring/	8646
17	exp Prenatal Diagnosis/	73597
18	exp Infant, Newborn/	603439
19	Pregnant Women/	8156
20	Infectious Disease Transmission, Vertical/	15756
21	Intensive Care Units, Neonatal/	14541
22	Intensive Care, Neonatal/	5628
23	(pregnan* or gestation* or parturition or neonatal* or neo natal* or neonate* or ante natal* or antenatal* or pre natal* or prenatal* or puerper* or postnatal* or postpartum or post partum or post natal* or peripartum or peri partum or intrapartum or intra partum or prepregnancy or pre pregnancy or preconception* or pre conception* or periconception* or peri conception* or preterm or premature or labo?r or eclamp* or preeclamp* or pre eclamp* or amniocentes* or chorion* vill* or breastfe* or breast fe* or lactation* or cesarean or caesarean or cesarian or caesarian or cesarien or caesarien or newborn* or new born* or tocoly* or fetal or foetal or fetus or foetus or miscarriage* or obstetric*).tw,kf,kw.	1430942

Supplementary Data 3: Data Items

Study Reference Identification Number
Author
Journal
Area covered (obstetrics, Maternal-fetal medicine, neonatology, fetal therapy etc.)
Changes to Low-risk antenatal clinics
Changes to High risk antenatal clinics (maternal/ Fetal/ Genetics/ ultrasound/ allied specialties)
Changes to Outpatient ambulatory care – day assessment units
Changes to Obstetric Triage
Changes to Postnatal ambulatory care or equivalent
Changes to Early pregnancy clinic
Changes to Screening and testing for COVID-19 in all areas
Changes to inpatient management - Antenatal ward
Changes to labour and childbirth (labour induction, elective and emergency caesareans, analgesia, those presenting in spontaneous labour
COVID-team
Changes to Fetal therapy
Changes to Postpartum (including ward care, early discharge, group classes etc)
Changes to Neonatal Intensive Care Unit Policies
Changes to Obstetric nursing
Changes to Perinatal mental health
Changes to Trainee schedules
Training of staff / PPE
Wellness initiatives
Anything specific with regard to clinical care - algorithms etc.
Other changes
Staff Restructuring
Visitor policies in various areas

24	((Vertical or Fetomaternal or Foetomaternal or Maternal-Fetal or Maternal Fetal or Maternal-Foetal or Maternal Foetal or Mother-To-Child or Mother to child) adj2 transmission*).tw,kf,kw.	12161
25	or/8-24	1976064
26	7 and 25	1666
27	26 and 20191101:20301231.(dt). [Create Date starting from November 2019]	474

Embase Translated Search:

Database(s): **Embase Classic+Embase** 1947 to 2020 May 14
Search Strategy:

#	Searches	Results
1	exp Coronavirinae/	14271
2	exp Coronavirus infection/	13034
3	(coronavirus* or corona virus* or corona virinae* or coronavirinae* or OC43 or NL63 or 229E or HKU1 or HCoV* or covid* or ncov* or coV or sars-cov* or sarscov* or Sars-coronavirus* or Severe Acute Respiratory Syndrome Coronavirus* or Severe Acute Respiratory Syndrome Corona virus* or 2019-ncov or 2019-novel CoV or SARS-like coronavirus*).mp.	37521
4	((novel or new or nouveau) adj2 pandemi*2).mp.	1055
5	((pneumonia or sars*).mp. or exp pneumonia/) and Wuhan.mp.	844
6	or/1-5	45280
7	exp Pregnancy/	798660
8	exp Pregnancy Disorder/	613094
9	exp Obstetric Procedure/	481868
10	pregnancy outcome/	58650
11	exp Breast Feeding/	54464
12	maternal health service/	1498
13	fetus/	213728
14	fetal therapy/	620
15	exp fetus monitoring/	14672
16	exp prenatal diagnosis/	110132
17	newborn/	614813
18	pregnant woman/	84823
19	neonatal intensive care unit/	9709
20	newborn intensive care/	26404
21	vertical transmission/	14787
22	(pregnan* or gestation* or parturition or neonatal* or neo natal* or neonate* or ante natal* or antenatal* or pre natal* or prenatal* or puerper* or postnatal* or postpartum or post partum or post natal* or peripartum or peri partum or intrapartum or intra partum or prepregnancy or pre pregnancy or preconception* or pre conception* or periconception* or peri conception* or preterm or premature or labo?r or eclamp* or preeclamp* or pre eclamp* or amniocentes* or chorion* vill* or breastfe* or breast fe* or lactation* or cesarean or caesarean or cesarian or caesarian or cesarien or caesarien or newborn* or new born* or tocoly* or fetal or foetal or fetus or foetus or miscarriage* or obstetric*).tw,kw.	1886202

23	((Vertical or Fetomaternal or Foetomaternal or Maternal-Fetal or Maternal Fetal or Maternal-Foetal or Maternal Foetal or Mother-To-Child or Mother to child) adj2 transmission*).tw,kw.	15286
24	or/7-23	2435123
25	6 and 24	2220
26	limit 25 to dc=20191101-20301231	467

Cochrane Translated Search:

Search Name: Covid19Pregnancy_RD_Cochrane_Final

Date Run: 15/05/2020 23:14:58

Comment: Limit Nov 2019 - Dec 2020

ID Search Hits

#1 MeSH descriptor: [Coronavirus] explode all trees (***13)***

#2 MeSH descriptor: [Coronavirus Infections] explode all trees (***131)***

#3 (coronavirus* or corona virus* or corona virinae* or coronavirinae* or OC43 or NL63 or 229E or HKU1 or HCoV* or covid* or ncov* or coV or sars-cov* or sarscov* or Sars-coronavirus* or Severe Acute Respiratory Syndrome Coronavirus* or 2019*ncov or 2019*novel CoV or SARS-like coronavirus*) (***1167)***

#4 ((novel or new or nouveau) NEAR/2 pandemi*) (***19)***

#5 (pneumonia or sars*) (***15874)***

#6 MeSH descriptor: [Pneumonia] explode all trees (***3504)***

#7 (Wuhan) (***1078)***

#8 (#5 OR #6) AND #7 (***63)***

#9 {OR #1-#4} OR #8 (***1208)***

#10 MeSH descriptor: [Pregnancy] explode all trees (***8451)***

#11 MeSH descriptor: [Pregnancy Complications] explode all trees (***11459)***

#12 MeSH descriptor: [Pregnancy Outcome] explode all trees (***3551)***

#13 MeSH descriptor: [Obstetrics] explode all trees (***178)***

#14 MeSH descriptor: [Breast Feeding] explode all trees (***1798)***

#15 MeSH descriptor: [Maternal Health Services] explode all trees (***2152)***

#16 MeSH descriptor: [Fetus] explode all trees (***1733)***

#17 MeSH descriptor: [Fetal Therapies] explode all trees (***37)***

#18 MeSH descriptor: [Fetal Monitoring] explode all trees (***360)***

#19 MeSH descriptor: [Prenatal Diagnosis] explode all trees (***803)***

#20 MeSH descriptor: [Infant, Newborn] explode all trees (***15549)***

#21 MeSH descriptor: [Pregnant Women] this term only (***245)***

#22 MeSH descriptor: [Infectious Disease Transmission, Vertical] this term only (***529)***

#23 MeSH descriptor: [Intensive Care Units, Neonatal] this term only (***712)***

#24 MeSH descriptor: [Intensive Care, Neonatal] this term only (***324)***

#25 (pregnan* or gestation* or parturition or neonatal* or neo natal* or neonate* or ante natal* or antenatal* or pre natal* or prenatal* or puerper* or postnatal* or postpartum or post partum or post natal* or peripartum or peri partum or intrapartum or intra partum or prepregnancy or pre pregnancy or preconception* or pre conception* or periconception* or peri conception* or preterm or premature or labo?r or eclamp* or preeclamp* or pre eclamp* or amniocentes* or chorion* vill* or breastfe* or breast fe* or lactation* or cesarean or caesarean or cesarian or caesarian or cesarien or caesarien or newborn* or new born* or tocoly* or fetal or foetal or fetus or foetus or miscarriage* or obstetric*):ti,ab,kw (***137436)***

#26 (((Vertical or Fetomaternal or Foetomaternal or Maternal-Fetal or Maternal Fetal or Maternal-Foetal or Maternal Foetal or Mother-To-Child or Mother to child) NEAR/2 (transmission*))):ti,ab,kw (***1994)***

#27 {OR #10-#26} (***138630)***

#28 #9 AND #27 (***150)***

#29 #9 AND #27 with Cochrane Library publication date Between Nov 2019 and Dec 2020 (***66)***

Cochrane Database of Systematic Reviews: ***(13) -Exported***
Cochrane Protocols: ***(2) – Exported (But because protocols are NOT published journal articles, they will not be included in the Systematic Review. Below is a screenshot of the 2 Protocols)***

Cochrane Reviews 13 | **Cochrane Protocols 2** | Trials 50 | Editorials 0 | Special collections 1 | Clinical Answers 0 | Other Reviews

2 Cochrane Protocols matching **"#29 - #9 AND #27" with Cochrane Library publication date Between Nov 2019 and Dec 2020**

Cochrane Database of Systematic Reviews
Issue 5 of 12, May 2020

Select all (2) Export selected citation(s) Hide all previews

Order by Relevancy Results per page 25

1 **Antidepressant treatment for postnatal depression**
Jennifer Valeska Elli Brown, Claire A Wilson, Karyn Ayre, Emily South, Emma Molyneaux, Kylee Trevillion, Louise M Howard, Hind Khalifeh
Intervention Protocol 20 March 2020 Free access
Hide preview
Abstract
This is a protocol for a Cochrane Review (Intervention). The objectives are as follows: To assess the effectiveness and safety of antidepressant drugs in comparison with any other treatment (psychological, psychosocial, or pharmacological), placebo, or treatment as usual for PND.

2 **High- versus low-dose conventional phototherapy for neonatal jaundice**
Yao Mun Choo, Shelley Springer, Ke Xin Yip, Azanna Ahmad Kamar, Eng Hwa Wong, Shaun Wen Huey Lee, Nai Ming Lai
Intervention Protocol 30 April 2020 Major change Free access
Hide preview
Abstract - Objectives
This is a protocol for a Cochrane Review (intervention). The objectives are as follows: To assess the effects of high-dose conventional phototherapy versus low-dose conventional phototherapy on bilirubin level and associated clinical outcomes that constitute the major conditions in the spectrum of…

Trials (Cochrane CENTRAL): ***(50)*** ***- Exported***
Editorials: (***0***)
Special collections: ***(1)*** ***– Special Collections cannot be exported & are not included in Systematic Reviews, see below for reference***
https://www-cochranelibrary-com.myaccess.library.utoronto.ca/collections/doi/SC000043/full

Cochrane Reviews	Cochrane Protocols	Trials	Editorials	Special collections	More
13	1	50	0	1	

1 Special collection matching **"#29 - #9 AND #27" with Cochrane Library publication date Between Nov 2019 and Dec 2020**

Hide all previews

Order by New To Old

Results per page 25

1

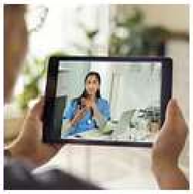

Coronavirus (COVID-19): remote care through telehealth

Hide preview 14 May 2020

The aim of this collection is to ensure immediate access to systematic reviews most directly relevant to remote health care through telehealth. The measures adopted internationally to curb the spread of COVID-19 have led to significant changes in how health care is accessed and provided.

CINAHL Translated Search:

Friday, May 15, 2020 4:41:01 PM

#	Query	Limiters/Expanders	Last Run Via	Results
S26	S6 AND S24	Limiters - Published Date: 20191101-20301231 Expanders - Apply equivalent subjects Search modes - Boolean/Phrase	Interface - EBSCOhost Research Databases Search Screen - Advanced Search Database - CINAHL Complete	91

S25	S6 AND S24	Expanders - Apply equivalent subjects Search modes - Boolean/Phrase	Interface - EBSCOhost Research Databases Search Screen - Advanced Search Database - CINAHL Complete	716
S24	S7 OR S8 OR S9 OR S10 OR S11 OR S12 OR S13 OR S14 OR S15 OR S16 OR S17 OR S18 OR S19 OR S20 OR S21 OR S22 OR S23	Expanders - Apply equivalent subjects Search modes - Boolean/Phrase	Interface - EBSCOhost Research Databases Search Screen - Advanced Search Database - CINAHL Complete	499,454
S23	TI (((Vertical or Fetomaternal or Foetomaternal or Maternal-Fetal or Maternal Fetal or Maternal-Foetal or Maternal Foetal or Mother-To-Child or Mother to child) N1 transmission*)) OR AB (((Vertical or Fetomaternal or Foetomaternal or Maternal-Fetal or Maternal Fetal or Maternal-Foetal or Maternal Foetal or Mother-To-Child or Mother to child) N1 transmission*)) OR MW (((Vertical or Fetomaternal or Foetomaternal or Maternal-Fetal or Maternal Fetal or Maternal-Foetal or Maternal Foetal or Mother-To-Child or Mother to child) N1 transmission*))	Expanders - Apply equivalent subjects Search modes - Boolean/Phrase	Interface - EBSCOhost Research Databases Search Screen - Advanced Search Database - CINAHL Complete	7,233
S22	TI ((pregnan* or gestation* or parturition or neonatal* or neo natal* or neonate* or ante natal* or antenatal* or pre natal* or prenatal* or puerper* or postnatal* or postpartum or post partum or post natal* or peripartum or peri partum or intrapartum or intra partum or prepregnancy or pre pregnancy or preconception* or pre conception* or periconception* or peri conception* or preterm	Expanders - Apply equivalent subjects Search modes - Boolean/Phrase	Interface - EBSCOhost Research Databases Search Screen - Advanced Search Database -	488,357

	or premature or labo?r or eclamp* or preeclamp* or pre eclamp* or amniocentes* or chorion* vill* or breastfe* or breast fe* or lactation* or cesarean or caesarean or cesarian or caesarian or cesarien or caesarien or newborn* or new born* or tocoly* or fetal or foetal or fetus or foetus or miscarriage* or obstetric*)) OR AB ((pregnan* or gestation* or parturition or neonatal* or neo natal* or neonate* or ante natal* or antenatal* or pre natal* or prenatal* or puerper* or postnatal* or postpartum or post partum or post natal* or peripartum or peri partum or intrapartum or intra partum or prepregnancy or pre pregnancy or preconception* or pre conception* or periconception* or peri conception* or preterm or premature or labo?r or eclamp* or preeclamp* or pre eclamp* or amniocentes* or chorion* vill* or breastfe* or breast fe* or lactation* or cesarean or caesarean or cesarian or caesarian or cesarien or caesarien or newborn* or new born* or tocoly* or fetal or foetal or fetus or foetus or miscarriage* or obstetric*)) OR MW ((pregnan* or gestation* or parturition or neonatal* or neo natal* or neonate* or ante natal* or antenatal* or pre natal* or prenatal* or puerper* or postnatal* or postpartum or post partum or post natal* or peripartum or peri partum or intrapartum or intra partum or prepregnancy or pre pregnancy or preconception* or pre conception* or periconception* or peri conception* or preterm or premature or labo?r or eclamp* or preeclamp* or pre eclamp* or amniocentes* or chorion* vill* or breastfe* or breast fe* or lactation* or cesarean or caesarean or cesarian or caesarian or cesarien or caesarien or newborn* or new born* or tocoly* or fetal or foetal or fetus or foetus or miscarriage* or obstetric*))		CINAHL Complete	
S21	(MH "Intensive Care, Neonatal")	Expanders - Apply equivalent subjects	Interface - EBSCOhost Research Databases	4,836

		Search modes - Boolean/Phrase	Search Screen - Advanced Search Database - CINAHL Complete	
S20	(MH "Intensive Care Units, Neonatal")	Expanders - Apply equivalent subjects Search modes - Boolean/Phrase	Interface - EBSCOhost Research Databases Search Screen - Advanced Search Database - CINAHL Complete	14,460
S19	(MH "Disease Transmission, Vertical")	Expanders - Apply equivalent subjects Search modes - Boolean/Phrase	Interface - EBSCOhost Research Databases Search Screen - Advanced Search Database - CINAHL Complete	6,214
S18	(MH "Expectant Mothers")	Expanders - Apply equivalent subjects Search modes - Boolean/Phrase	Interface - EBSCOhost Research Databases Search Screen - Advanced Search Database - CINAHL Complete	8,244
S17	(MH "Obstetric Care+")	Expanders - Apply equivalent subjects Search modes - Boolean/Phrase	Interface - EBSCOhost Research Databases	53,782

			Search Screen - Advanced Search Database - CINAHL Complete	
S12	(MH "Maternal Health Services+")	Expanders - Apply equivalent subjects Search modes - Boolean/Phrase	Interface - EBSCOhost Research Databases Search Screen - Advanced Search Database - CINAHL Complete	32,595
S11	(MH "Breast Feeding+")	Expanders - Apply equivalent subjects Search modes - Boolean/Phrase	Interface - EBSCOhost Research Databases Search Screen - Advanced Search Database - CINAHL Complete	25,010
S10	(MH "Obstetrics")	Expanders - Apply equivalent subjects Search modes - Boolean/Phrase	Interface - EBSCOhost Research Databases Search Screen - Advanced Search Database - CINAHL Complete	6,082
S9	(MH "Pregnancy Outcomes")	Expanders - Apply equivalent subjects Search modes - Boolean/Phrase	Interface - EBSCOhost Research Databases	24,787

			Search Screen - Advanced Search Database - CINAHL Complete	
S8	(MH "Pregnancy Complications+")	Expanders - Apply equivalent subjects Search modes - Boolean/Phrase	Interface - EBSCOhost Research Databases Search Screen - Advanced Search Database - CINAHL Complete	97,903
S7	(MH "Pregnancy+")	Expanders - Apply equivalent subjects Search modes - Boolean/Phrase	Interface - EBSCOhost Research Databases Search Screen - Advanced Search Database - CINAHL Complete	223,346
S6	S1 OR S2 OR S3 OR S4 OR S5	Expanders - Apply equivalent subjects Search modes - Boolean/Phrase	Interface - EBSCOhost Research Databases Search Screen - Advanced Search Database - CINAHL Complete	17,643
S5	TX (((pneumonia or sars*) OR (MH "Pneumonia+")) AND (TX (Wuhan)))	Expanders - Apply equivalent subjects Search modes - Boolean/Phrase	Interface - EBSCOhost Research Databases	478

			Search Screen - Advanced Search Database - CINAHL Complete	
S4	TX ((novel or new or nouveau) N1 pandemi*)	Expanders - Apply equivalent subjects Search modes - Boolean/Phrase	Interface - EBSCOhost Research Databases Search Screen - Advanced Search Database - CINAHL Complete	636
S3	TX (coronavirus* or corona virus* or corona virinae* or coronavirinae* or OC43 or NL63 or 229E or HKU1 or HCoV* or covid* or ncov* or coV or sars-cov* or sarscov* or Sars-coronavirus* or Severe Acute Respiratory Syndrome Coronavirus* or 2019-ncov or 2019-novel CoV or SARS-like coronavirus*)	Expanders - Apply equivalent subjects Search modes - Boolean/Phrase	Interface - EBSCOhost Research Databases Search Screen - Advanced Search Database - CINAHL Complete	15,112
S2	(MH "Coronavirus Infections+")	Expanders - Apply equivalent subjects Search modes - Boolean/Phrase	Interface - EBSCOhost Research Databases Search Screen - Advanced Search Database - CINAHL Complete	3,248
S1	(MH "Coronavirus+")	Expanders - Apply equivalent subjects Search modes - Boolean/Phrase	Interface - EBSCOhost Research Databases	871

			Search Screen - Advanced Search Database - CINAHL Complete	
S16	(MH "Infant, Newborn+")	Expanders - Apply equivalent subjects Search modes - Boolean/Phrase	Interface - EBSCOhost Research Databases Search Screen - Advanced Search Database - CINAHL Complete	145,151
S15	(MH "Prenatal Diagnosis+")	Expanders - Apply equivalent subjects Search modes - Boolean/Phrase	Interface - EBSCOhost Research Databases Search Screen - Advanced Search Database - CINAHL Complete	20,315
S14	(MH "Fetal Monitoring+")	Expanders - Apply equivalent subjects Search modes - Boolean/Phrase	Interface - EBSCOhost Research Databases Search Screen - Advanced Search Database - CINAHL Complete	3,261
S13	(MH "Fetus+")	Expanders - Apply equivalent subjects Search modes - Boolean/Phrase	Interface - EBSCOhost Research Databases	28,635

			Search Screen - Advanced Search Database - CINAHL Complete	

Scopus Translated Search:

((ALL (coronavirus* OR "corona virus" OR "corona viruses" OR "corona virinae" OR coronavirinae* OR oc43 OR nl63 OR 229e OR hku1 OR hcov* OR covid* OR ncov* OR cov OR sars-cov* OR sarscov* OR sars-coronavirus*) OR ALL ("Severe Acute Respiratory Syndrome Coronavirus" OR "Severe Acute Respiratory Syndrome Coronaviruses" OR 2019-ncov OR "SARS-like coronavirus" OR "SARS-like coronaviruses")) OR (ALL (((novel OR new OR nouveau) W/2 pandemi*))) OR (ALL (((pneumonia OR sars*) AND wuhan)))) AND ((TITLE-ABS-KEY (pregnan* OR gestation* OR parturition OR neonatal* OR "neo natal" OR neonate* OR antenatal* OR "ante natal" OR prenatal* OR "pre natal" OR puerper* OR postnatal* OR "post natal" OR postpartum OR "post partum" OR peripartum OR "peri partum" OR intrapartum OR "intra partum" OR prepregnancy OR "pre pregnancy" OR preconception* OR "pre conception" OR periconception* OR "peri conception" OR preterm OR premature OR labo?r OR eclamp* OR preeclamp* OR "pre eclampsia" OR amniocentes* OR chorion* AND vill* OR breastfe* OR "breast fed" OR "breast feeding" OR lactation* OR cesarean OR caesarean OR cesarian OR caesarian OR cesarien OR caesarien OR newborn* OR "new born" OR "new borns" OR tocoly* OR fetal OR foetal OR fetus OR foetus OR miscarriage* OR obstetric*)) OR (TITLE-ABS-KEY ((vertical OR fetomaternal OR foetomaternal OR maternal-fetal OR "Maternal Fetal" OR maternal-foetal OR "Maternal Foetal" OR mother-to-child OR "Mother to child") W/2 (transmission*)))) AND (LIMIT-TO (PUBYEAR , 2020) OR LIMIT-TO (PUBYEAR , 2019))

282 Document Results

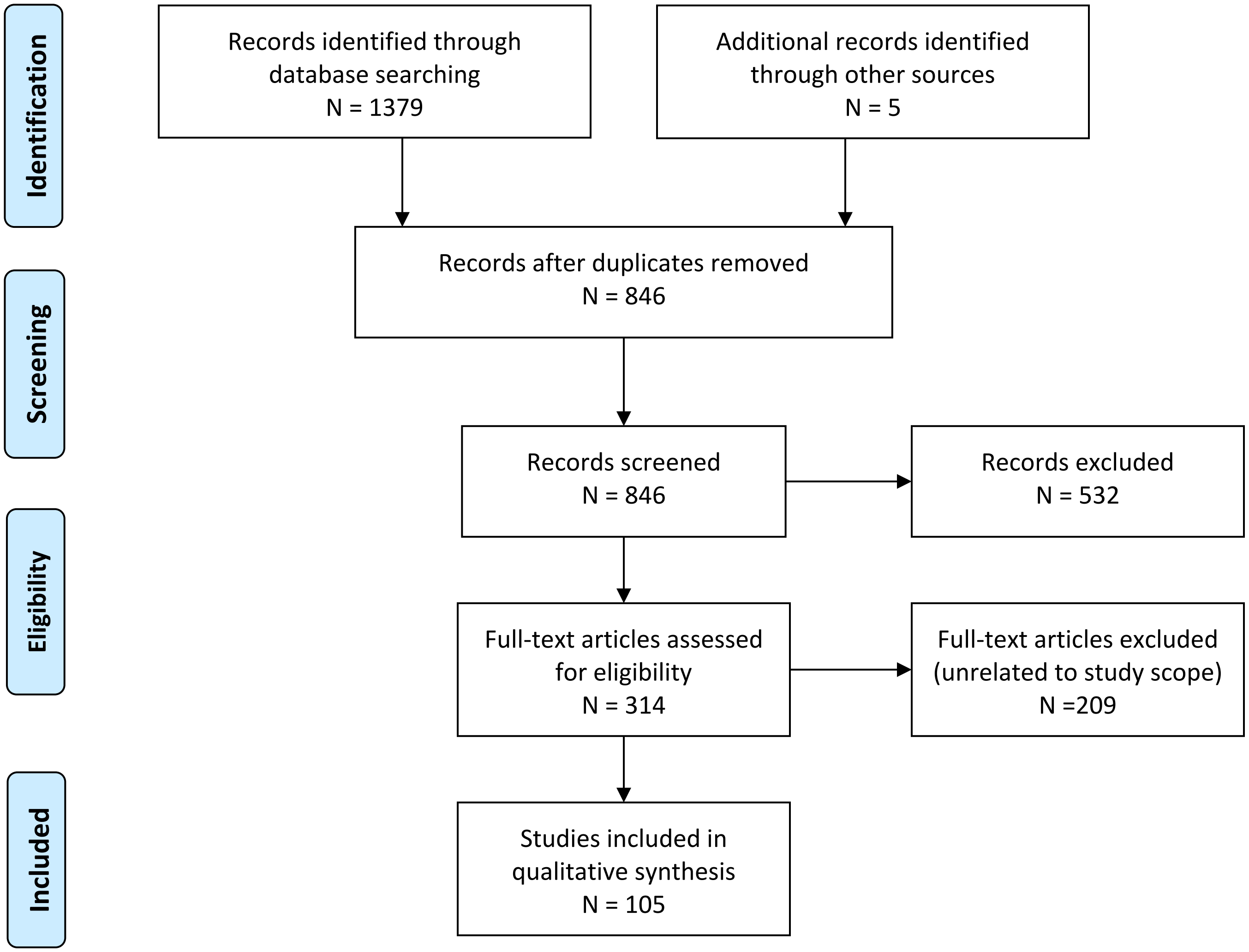
Identification
Screening
Eligibility
Included
Records identified through database searching N = 1379
Additional records identified through other sources N = 5
Records after duplicates removed N = 846
Records screened N = 846
Records excluded N = 532
Full-text articles assessed for eligibility N = 314
Full-text articles excluded (unrelated to study scope) N =209
Studies included in qualitative synthesis N = 105

Ambulatory Visit of a Confirmed or Suspected Case of COVID-19

If washroom is needed, escort patient to designated washroom.

Washroom is locked with a sign "STOP, Do Not Enter".

After use, page service assistant: to complete terminal clean x2

Give written instructions to patient : 1. Meeting point. 2 How to don mask

Patient to follow Public Health guidelines for travel to clinic

On arrival, Patient calls Ambulatory Clinical Co-Ordinator. *If* no answer, call Clinical Director

Patient waits at meeting point until greeted by staff member

Clinical co-ordinator/ Clinical director will notify clerk at relevant clinic to check patient in

Notify healthcare provider of patients arrival. Healthcare provider to don droplet/contact PPE

Proceed to meeting point to greet patient and give them a mask (if they do not already have one)

Patient to don mask & perform hand hygiene, before entering elevator/ clinic area

Enter clinic area through designated door to avoid unnecessary staff/patient contact

Take patient to ultrasound room or consult room as required

Place Droplet Contact sign on door. Do <u>NOT</u> bring paper chart into room

Give patient their follow-up appointment(s) before leaving room

After the appointment is completed: 1. Doff all PPE <u>inside</u> the room, *except* mask. 2. Escort patient to elevators and advise them to immediately exit building 3. Put "do not enter" sign on room door

Page service assistant to complete Terminal Clean x 2 after patient leaves room/washroom

<u>Staff check list:</u>

- Ensure Appointment room is stocked
- Ensure PPE cart is stocked and placed outside appointment room
- Review chart to determine if any lab requisitions or specimens need completion

COVID-19 Antenatal Care Clinic

Background

- During the months of April, May and June 2020 we anticipate increased volumes of COVID-19 positive pregnant patients who are outpatients requiring antenatal visits and fetal ultrasound
- COVID-19 poses increased pregnancy risk based on emerging data on pregnancy outcome, thus increased fetal surveillance may be indicated (fetal well being, fetal growth, possibly maternal cervical length)
- As the low- and high-risk pregnancy clinics are relatively busy areas (clinical activity has been ramped down), having multiple COVID positive patients in the environment may pose risk to other patients and care providers. Having separate location + dedicated team may reduce overall exposure
- Although difficult to predict exactly how many patients will need this service, it would be reasonable to implement the plan if our COVID positive population reaches 5-10 patients per week.

RN and MD staffing clinic to don appropriate PPE for Clinic and present to the 2nd floor

- PPE to be brought over with them from OPG
- BP cuff to be provided by WCH
- No paper charts; no Doptone transported over

Antenatal patient known COVID-positive status requires antenatal visit and/or fetal ultrasound

Patient presents to side entrance of WCH and takes dedicated elevator to 2nd floor Waiting area

- Greeted by WCH Navigator
- Performs hand hygiene
- Puts on mask
- Patient directed to the Assessment Room or US Room

RN to perform assessment

- Weight, blood pressure
- Record in Electronic Medical Records
- Guide patient to door
- Navigator guides patient to US room

Room cleaning to commence

MD to perform fetal assessment

- US as appropriate for gestational weeks and pregnancy complications
- Complete assessment and document in Electronic Medical Records
- Patient directed to waiting area

Room cleaning to commence)

Patient visit complete

Navigator directs patient to perform hand hygiene and guides patient to the exit

RN/MD to email SPP admin patient list with orders for each patient and to indicate timing of next visit and/or US

Supplementary Data 7

Low-Risk Inclusion Criteria for Early Discharge, less than 24 hours after birth

24-36 Hour post birth follow-up must be in place for newborn assessment/ screening tests.

Mother	Newborn
• Willing to receive follow-up care at designated location<br>• Vaginal delivery<br>• Has voided independently<br>• If GDM: protocol complete<br>• Perineal repair: 2nd degree or less<br>• Vital signs stable with normal postpartum assessment (lochia, fundus)<br>• Stable mood with psychosocial support and no concerns with home environment<br>• No pre-existing hypertension, PPH, gestational hypertension, chorioamnionitis, intrapartum complication or other medical concern(s)<br>• Required MMR, RhoGam and medications have been administered<br>• Mother/guardian demonstrates satisfactory knowledge and comprehension for self-care and newborn care including how to access care in an emergency<br>• MRP (or delegate) has written an order for early discharge	• Primary health care provider in the community has been established<br>• 37 weeks' gestation or greater<br>• If mother GBS positive, adequate intrapartum prophylaxis provided<br>• If assisted vaginal delivery: discharge after 12hrs<br>• APGAR at 5 minutes: 7 or higher<br>• Newborn exam completed by MRP<br>• DAT negative<br>• Hypoglycemia protocol complete is indicated<br>• No SGA<br>• No risk factors for sepsis<br>• If breastfeeding, one successful latch has been observed. If bottle feeding, skill with bottle feeding has been observed.<br>• Vital signs are stable<br>• Newborn standards of care have been met (Vitamin K, Erythromycin prophylaxis if indicated)<br>• Required immunizations (e.g. Hepatitis) have been administered<br>• MRP has written an order for early discharge
